# FOOPAS Study: Functional Assessment and Prognostic Value in Aortic Valve Replacement for Patients ≥ 75 Years

**DOI:** 10.3390/jcm15103750

**Published:** 2026-05-13

**Authors:** Dennis Eckner, Susanne Wicklein, Markus Gosch, Theodor Fischlein, Basel Habboub, Jürgen Jessl, Matthias Pauschinger, Ferdinand Vogt

**Affiliations:** 1 Department of Cardiology, Klinikum Nürnberg Paracelsus Medical University Nuremberg, 90471 Nuremberg, Germany; dennis.eckner@klinikum-nuernberg.de (D.E.); juergen.jessl@klinikum-nuernberg.de (J.J.); matthias.pauschinger@klinikum-nuernberg.de (M.P.); 2 Department of Geriatric Medicine, Paracelsus Medical University Nuremberg, 90419 Nuremberg, Germany; susanne.wicklein@klinikum-nuernberg.de (S.W.); markus.gosch@klinikum-nuernberg.de (M.G.); basel.habboub@klinikum-nuernberg.de (B.H.); 3 Department of Cardiac Surgery, Paracelsus Medical University Nuremberg, 90419 Nurnberg, Germany; theodor.fischlein@klinikum-nuernberg.de

**Keywords:** aortic stenosis (AS), surgical aortic valve replacement (SAVR), transcatheter aortic valve replacement (TAVR), geriatric assessment, risk stratification

## Abstract

**Background**: Because of demographic changes, the number of older patients undergoing cardiac interventions has increased. The most common indication in this group is aortic valve stenosis, treated with either surgical aortic valve replacement (SAVR) or transcatheter aortic valve implantation (TAVR), with good outcomes. Our study investigated whether the heart team’s choice of intervention (TAVI, SAVR, or conservative) is influenced by geriatric assessment results. **Methods:** This study was a single-centre, prospective, longitudinal case–control study conducted over 12 months and did not affect routine diagnostic examinations or clinical decisions. After risk stratification and clinical evaluation, patients were assigned to undergo TAVI, SAVR, or conservative management. Cardiological evaluation and geriatric assessment were performed for up to 12 months. **Results:** Of 135 patients (mean age 81 ± 4.6 years), 60% underwent TAVI, 29% SAVR, and 11% conservative therapy. Age, Frailty Score, cognition, and nutritional status were significantly associated with the heart team’s decision, whereas EuroSCORE II remained the only independent predictor of one-year mortality (OR 1.58, 95% CI 1.13–2.19, *p* = 0.007). One-year mortality was 9.9% (*n* = 11). Compared to the literature, one-year mortality was lower than expected, particularly in the intervention group. **Conclusions:** Single assessment tools did not have the power to predict mortality. Similar to other trials, a combination of different scores can assess the risk of mortality.

## 1. Introduction

Demographic changes have increased old and very old patients requiring heart surgery or cardiological interventions. The most frequent indication for this group of patients is aortic valve stenosis (AS). In contrast, conservative management results in a significantly worse outcome; surgical aortic valve replacement (SAVR) and transcatheter aortic valve implantation (TAVI) are established treatment options, even in very old patients. Both SAVR and TAVI result in a good outcome among older adults in terms of mortality [1,2,3].

Studies on aortic valve replacement (AVR) in older adults have shown excellent results, especially regarding excess mortality. These results contrast with clinical experience, especially if mortality is not the only outcome parameter. Nowadays, decisions regarding AVR procedures are mainly driven by age, cardiovascular parameters, and an empirical assessment of surgical risk. They do not include a standardized geriatric assessment of the functional status of a patient. Standardized integration of geriatric assessment has been limited by a lack of consensus on methodology and has therefore not yet been implemented in clinical practice. In geriatric assessment, functional status should be assessed using validated tests regardless of the diagnosis. In clinical settings such as orthogeriatric care, various geriatric assessment tools have demonstrated the ability to predict clinical outcomes [4,5].

Frailty is a geriatric syndrome that influences potential functional recovery after TAVI or SAVR [6,7]. Frailty is understood as a diminished capability to recover from pathological or iatrogenic stressors due to ageing-related impairments [8]. Although the likelihood of short-term procedural success exceeds 95% [9], two out of five patients in the PARTNER (Placement of Aortic Transcatheter Valves) I and CoreValve pivotal trials experienced poor health-related quality of life or death over the ensuing year [10].

Currently, “frailty” is an additional criterion that is roughly assessed by “eyeballing”, based on the surgeon’s or interventionalist’s experience.

In the FOOPAS study (Functionality and Outcome in Older Patients with severe Aortic Stenosis), we investigated, in a prospective design, the integration of a comprehensive geriatric assessment independent of the heart team’s decision. This allows us to directly compare geriatric parameters with established cardiac risk scores. This study focuses on the link between geriatric domains and treatment allocation, and not just outcome prediction, such as age or EuroSCORE II.

## 2. Materials and Methods

### 2.1. Patients

Between May 2017 and August 2020, patients aged 75 years or older with symptomatic AS were screened at the Klinikum Nuremberg. A total of 679 SAVRs were performed: 452 isolated procedures and 227 combination procedures. In addition, 675 transfemoral and 109 transapical TAVIs were performed. Patient recruitment was based on an “all-comers” principle. Recruitment was limited both by strict exclusion criteria and by reduced enrollment during the COVID-19 pandemic, yielding 135 patients. All patients underwent an interdisciplinary heart team evaluation and were enrolled in a single-centre, prospective, longitudinal case–control study over 12 months. Exclusion criteria included missing patient approval, combined aortic valve disease, history of heart surgery, acute coronary syndrome within four weeks, and a limited life expectancy of approximately 12 months. Following the decision of the heart team (cardiologists and cardiac surgeons), further treatment was determined.

After the heart team’s decision, based on risk stratification and clinical evaluation, patients were assigned to undergo SAVR, TAVI, or conservative, optimized medical treatment. Cardiological parameters were assessed by the heart team following routine clinical standards and complemented by the parameters of the PARTNER 2A trial [11]. Regardless of the therapeutic approach, all patients received a comprehensive geriatric assessment and a consultation with a geriatrician, with individualized recommendations (e.g., medication adjustments, nutrition recommendations, fall prevention). In addition, all groups received guideline-directed medication (β-blockers, ACE/ARB, diuretics, and anticoagulation where indicated). The results of the geriatric assessment did not affect the treatment decision. Follow-ups and further assessments were scheduled before discharge (day three to five), and at three and 12 months after inclusion.

The study was registered in the German Clinical Trials Register (DRKS00012494) and approved by the ethical review board of the Bavarian Medical Association (Protocol Number 17011).

We collected demographic data (age, gender), echocardiographic parameters (aortic valve area, aortic valve gradient [maximum and mean], left ventricular ejection fraction), parameters from the clinical presentation (New York Heart Association [NYHA] Class I–IV, systolic and diastolic blood pressure, heart rate, respiratory rate, oxygen saturation, and body mass index [BMI]), laboratory tests (hemoglobin, sodium, glomerular filtration rate, N-terminal prohormone of brain natriuretic peptide [NT-proBNP], C-reactive protein [CRP], and albumin) and calculated the EuroSCORE II and Society of Thoracic Surgeons (STS) score.

Baseline characteristics are shown in Table 1.

To assess the level of social support, we collected information on the patient’s place of residence, a marker of independence and autonomy. In order to evaluate and analyze comorbidities, we applied two different scores. First, the Charlson Comorbidity Index (CCI), which includes 17 items [12], is a valuable tool to predict 1-year mortality in older adults. Each condition is assigned a score of 1, 2, 3, or 6 according to the risk of death, and the total score predicts mortality. Second, the Modified Cumulative Illness Rating Scale (CIRS) [13] is a marker of multimorbidity. Each organ system is assessed on a scale of 0 to 4. Polypharmacy was evaluated by summing the number of medications.

For quality of life, we used the health questionnaire EuroQol-5D [14], which assesses five items (mobility, self-care, usual activities, pain/discomfort, and anxiety/depression) using a rating scale from 0 (worst) to 100 (best).

Frailty was assessed using the frailty criteria defined by Fried. Each item was allocated 1 point, with total scores ranging from 0 to 5 [15]. Functionality in Activities of Daily Living was recorded using the Activities of Daily Living (ADL) and Instrumental Activities of Daily Living (IADL) scores [16,17]. The ADL score is a valid tool for assessing overall function in daily living in relation to 10 items (bowels, bladder, grooming, toilet use, feeding, transfer, mobility, dressing, stairs, and bathing). The IADL scale of Lawton and Brody is a similar tool that assesses the everyday competence of older adults and contains eight central activities for daily living and autonomy (ability to use a telephone, shopping, food preparation, housekeeping, laundry, mode of transportation, responsibility for own medication, ability to handle finances). For psychometric evaluation, we used the Mini-Mental State Examination (MMSE) and Geriatric Depression Scale (GDS) [18,19]. Mobility was assessed by the Parker Mobility Score (PMS), the Timed Up&Go (TUG) test, and gait speed. The PMS assesses mobility in three situations (in the house, outside, and shopping); it is well established in orthogeriatric trials and has shown a strong correlation with short- and long-term mortality [20]. The TUG assesses gait speed, gait disabilities, and muscle strength by measuring the time, in seconds, it takes to get up from a chair, walk 3 m, turn around, and sit down [21]. The TUG test is very useful for observing the course of treatment or rehabilitation. Gait speed is simple to measure and an objective parameter, with reduced speed predicting worse outcomes in older adults. Gait speed is typically measured over a distance of 6 m; the cut-off is 0.8 m/s [22].

Handgrip strength is a valid test for estimating muscle strength in older adults. Handgrip strength was measured using a vigorimeter [23]. We used the Mini-Nutritional Assessment Short Form (MNA-SF) to evaluate nutrition status [24]. All geriatric assessments were carried out by specialized study nurses.

To compare the groups, we also performed a Global Fit Score (GFS). This score included frailty (Frailty Score ≤ 1), multimorbidity (CCI ≤ 2), mobility (PMS ≥ 8), cognition (MMSE > 27), and nutrition (MNA-SF > 11). Each feature was allocated 1 point, with 5 points corresponding to the fittest patients and 0 to the frailest.

**Figure 1:**
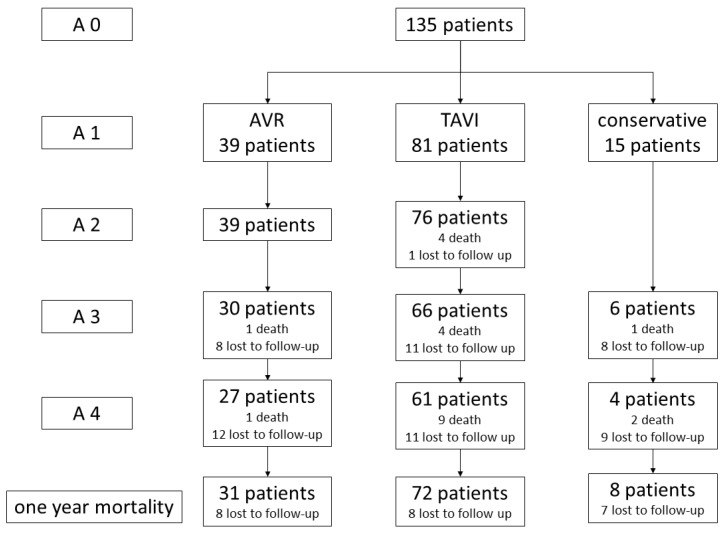
Consort diagram.

The study chart, including the number of patients, is shown in Figure 1.

The routine cardiological examinations conducted during the process of treatment allocation and pretesting were documented as test battery A0. A0 included no special geriatric parameter because, at this stage, geriatric evaluation did not play any role. Test battery A1 was performed after the indication, and the decision about the treatment approach was made by the heart team. Test battery A1 focused on geriatric aspects and geriatric assessment. Test battery A2 was a postinterventional course and was limited to the hospital stay, particularly the perioperative phase. Test battery A3 was scheduled three months after inclusion. Test battery A4 focused on outcomes after 12 months. Details of the study design were published in 2017 [25].

### 2.2. Statistics

An identification number was allocated to each patient. Data collection was carried out by study nurses and data were entered into a Microsoft Access 2016 database. For statistical analysis, all data were transferred to SPSS Statistics for Windows, Version 22.0. Data consistency was checked. Distributions were evaluated for normal distribution based on Kolmogorov–Smirnov tests. Categorical data are shown as absolute numbers and percentages, and all continuous data as median, standard deviation, and range. *p*-values were calculated using Fisher’s exact test (for categorical data), Mann–Whitney U test, and Kruskali–Wallis test (for comparing median values). All tests were two-sided, with the significance level set to α = 0.05.

The primary analysis explored the association between geriatric assessments at baseline and each of the two outcomes: one-year all-cause mortality and treatment decision. Multivariate logistic regression analyses were performed to identify factors associated with the treatment decision of the heart team and one-year mortality. The results are presented as odds ratios (ORs) with 95% confidence intervals (CIs).

The first model included the treatment decision (TAVI vs. SAVR) as the dependent variable. Due to the small number of patients that underwent conservative treatment (*n* = 15), this group was not included in the logistic regression analysis. The independent variables were age, EuroSCORE II, and the geriatric assessment (CIRS, CCI, EuroQol-visual analogue scale [EQ-VAS], ADL, IADL, Frailty Score, MMSE, GDS, Gaitspeed, PMS, handgrip, MNA-SF).

The second model included one-year all-cause mortality as the dependent variable. The independent variables were age, EuroSCORE II, and all variables of the geriatric assessment as outlined above. Due to mortality being a rare event in our study (9.9%), ORs calculated through multivariate logistic regression were an appropriate estimate of relative risk.

Secondary analysis:

In the secondary analysis, we explored the factors associated with quality of life (QoL), which were assessed using the EQ-VAS. Because of the small number of patients in the conservative group after 12 months (*n* = 8), only patients who underwent TAVI or SAVR were included in the analysis. Significant improvement in QoL after 12 months was defined as an increase of at least 10 points, and the continuous EQ-VAS difference was transformed into a binary outcome (increase in QoL vs. no increase in QoL). We compared the geriatric assessment for both groups using Fisher’s exact test (for categorical data) or the Mann–Whitney U test.

In a post hoc analysis, the effect of all three treatments on one-year all-cause mortality was compared using Fisher’s exact test.

One-year analyses were based on complete cases; the potential for selective dropout bias cannot be excluded.

## 3. Results

A total of 135 patients were included in the analysis, of whom 81 (60%) underwent TAVI, 39 (28.9%) underwent SAVR, and 15 (11.1%) received conservative treatment. One-year follow-up data were available for 111 patients, 64 in the TAVI group, 31 in the SAVR group, and 8 in the conservative group.

Echocardiographic parameters showed no significant differences between treatment groups. As expected, patients in the TAVI and conservative treatment groups were significantly older compared with those in the SAVR group. No gender differences were observed, but higher NYHA class was observed in the conservative group. Regarding vital signs, patients in the SAVR group had higher systolic blood pressure than those in the other two groups, while the other vital signs showed no significant differences.

Regarding laboratory tests, significantly worse values were found in the SAVR and conservative groups, except for sodium and CRP. Apparent differences were found in both scores used to assess perioperative mortality risk (EuroSCORE II and STS) regarding the different treatment arms. Table 1 shows baseline values for vital signs, cardiological parameters, and laboratory tests.

As shown in Table 2, significant differences were found in all geriatric assessment tests, except the Geriatric Depression Scale and medication.

The multivariate analysis showed that age, Frailty Score, cognition, and nutritional status were significantly correlated with the heart team’s decision (Table 3).

**Figure 2:**
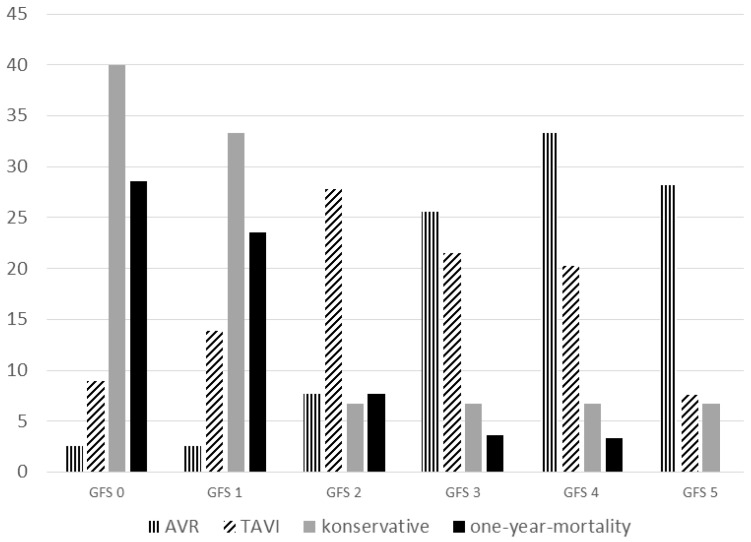
Global Fit Score, treatment groups, and one-year mortality (percentage).

The three treatment arms showed apparent differences in GFS, and there was also a significant correlation (*p* = 0.001, correlation coefficient 0.293) between GFS and one-year mortality (Figure 2).

Information on short-term mortality was available for all patients. Within 30 days, two (2.6%) patients died, one in the TAVI group and one in the SAVR group. One-year mortality data was available for 111 patients: 8 had conservative treatment, 31 underwent SAVR, and 72 underwent TAVI. Overall, one-year mortality was 9.9% (*n* = 11). The highest mortality was among patients receiving conservative treatment (25.0%), followed by TAVI patients (11.1%) and SAVR patients (3.2%) (Table A1). Significant differences were found in the maximum pressure gradient, blood pressure, BMI, and laboratory parameters, except for sodium and CRP. The risk scores were significantly associated with one-year mortality.

EuroSCORE II, *p* < 0.0001, and STS-Score, *p* = 0.002. The geriatric tests and scores showed significant differences between all procedures except CIRS, GDS, and handgrip strength.

In multivariate analyses, EuroSCORE II remained the only independent predictor of mortality (Table 4).

In total, 7.4% of the TAVI group had no functional deficits. Also in this group, 67% of the patients had no cognitive impairment (MMSE > 27), 63.3% had no pronounced multimorbidity (CCI ≤ 2), 54.3% had good nutritional status (MNA-SF > 11), 53% had no relevant mobility impairment (PMS ≥ 8), and 46.9% were fit (Frailty Score ≤ 1).

In addition, we investigated potential associations between geriatric parameters and QoL (*n* = 88). Only the baseline EQ-VAS was significantly associated with QoL changes after the intervention (Table 5).

No conclusions could be drawn about the remaining geriatric parameters and QoL after the intervention (Table A2 and Table A3).

## 4. Discussion

Our prospective study evaluated (1) whether treatment decisions by heart teams were influenced by the results of geriatric assessments in older patients; and (2) the predictive value of the various assessment tools. In our study, patients in the TAVI group had a significantly higher degree of functional impairment than patients who received SAVR. However, in multivariable analysis, in addition to age, only cognitive impairment, frailty, and poor nutritional status were significant predictors of treatment allocation. This indicates that treatment decisions are primarily based on rapid visual assessment (“eyeballing”) by the heart team. Our data did not yield a single geriatric score that was associated with the treatment decision. Nevertheless, a comprehensive geriatric assessment may still facilitate improved management and better outcomes.

In this cohort, one-year mortality was substantially lower than previously reported. Predictors of mortality—including frailty, nutritional status, cognition, and age—suggest that treatment decisions are frequently guided by the heart team’s overall clinical impression, known as “eyeballing”, rather than by standardized assessment tools [26]. The excellent outcomes in the included patient population leaves room for interpretation. On the one hand, the heart team’s experience could have contributed to this low mortality rate. On the other hand, there may also be a selection bias toward fitter patients. Despite various limitations, FOOPAS provides significant results and shows research deficits. In particular, the extent to which geriatric assessment procedures can support the decision to perform aortic valve replacement in patients with severe aortic stenosis remains unclear.

Our findings suggest that geriatric scores and the geriatric perspective are virtually neglected when deciding the most appropriate treatment approach. Of course, older patients have a higher level of functional impairment, but age alone cannot be the main criterion for treatment decisions. Unfortunately, based on our data and the study design, we cannot conclude which geriatric scores should be used for treatment decisions. The general trend is currently towards TAVI even in younger patients, but the question remains whether certain patient groups might also benefit from SAVR in old age.

In the TAVI group, 46.9% were fit (Frailty Score ≤ 1), 53% had no relevant mobility impairment (PMS ≥ 8), 67% had no cognitive impairment (MMSE > 27), 54.3% had good nutritional status (MNA-SF > 11), and 63.3% had no pronounced multimorbidity (CCI ≤ 2). Based on these criteria, 7.4% of the TAVI group had no functional deficits. Against this background, individual assessment of each older patient before AVR is crucial [26].

The primary outcome in the study was one-year mortality. In the overall study population, one-year mortality was 11%, regardless of treatment approach. As expected, the highest mortality rate was found in the non-intervention group. In the intervention group, mortality was 8.7%, 3.2% (SAVR) vs. 11.1% (TAVR).

Compared to international data, one-year mortality was significantly lower in both intervention groups. Kundi et al. demonstrated one-year mortality of 17–18.6% for AVR and 17.5–19.4% for TAVI patients [27]. It has been shown previously that EuroSCORE II may overestimate 30-day mortality [28]. The predominance of EuroSCORE II in the multivariable model likely reflects that this composite index encapsulates multiple clinical risk domains already considered by the heart team and may partially overlap with frailty and comorbidity measures. Among our patients, one patient died in the TAVI group and one in the SAVR group.

Frailty as a syndrome has become increasingly crucial in cardiac surgery and cardiology in recent years. Allen aptly titled his 2014 editorial in the *Journal of Thoracic and Cardiovascular Surgery*, “Frailty; It’s hard to define, but you know it when you see it.” At the same time, he called for moving away from eyeballing by surgeons and cardiologists toward a standardized test for frailty [28]. In FOOPAS, frailty, as measured by Fried’s criteria, had a significant influence on the choice of intervention (*p* = 0.044) and on one-year mortality (*p* = 0.002) in older patients. The effect on mortality was also described in a review by Sepehri, from 2014 [29]. For TAVI, too, a significant impact of frailty on the outcome has been demonstrated [30]. Based on data from the Frailty-AVR study, the guidelines currently cite the “Essential Frailty Toolset” (EFT) [26,31]. The FOOPAS study concept was developed before 2017. The chair-rising test in the EFT was assessed by our study group as potentially problematic or risky, especially in patients with hemodynamically relevant AS. The GFS calculated in FOOPAS showed a good correlation with both the choice of intervention and the outcome. Our GFS included frailty, multimorbidity, mobility, nutrition, and cognition. These components are well established as geriatric assessment tools. Frailty should therefore not only be used for the assessment of prognosis, but also for the choice of AVR modality. The optimal tool for assessing frailty requires further study and, particularly, which method can meaningfully assist with the selection of intervention.

The other assessments collected, aside from the GDS, also showed significant differences between the groups. As mentioned previously, we did not use the chair-rising test to assess muscle strength and mobility, but TUG, gait speed, PMS, and grip strength. All parameters showed significant differences between groups. In particular, the PMS could be a valuable alternative for clinical practice as a simple anamnestic tool. In multivariable analysis, only age, MMSE, Frailty Score, and MNA-SF were significantly associated with the decision between TAVI and SAVR. All these parameters can undoubtedly be explained in the context of the “eyeballing” effect of the heart team.

Almost similar results were found concerning one-year mortality. After multivariable analysis, EuroSCORE II remained the only independent predictor. This result is consistent with previous studies, indicating that a combination of different tools is valuable and necessary for risk assessment [32]. Single parameters should not be used to evaluate prognosis in older patients with AS.

Apart from mortality, QoL is crucial and, from the patient’s perspective, may even be more important than mortality. Several studies have already demonstrated positive effects of TAVI on QoL, including in older adults [33,34,35]. As a measure of improvement in QoL, an increase of ≥10 points in the EQ-VAS has been used. Our data showed that patients with poor QoL derive a particular benefit from intervention. Similar to the German Aortic Valve Registry (GARY), patients with a low baseline EQ-5D had a significant improvement in QoL one year after TAVI [36]. All other parameters had no predictive value with regard to which patient benefits from which intervention. Contrary to Stanska et al., there was no difference in our collective between the TAVI and the AVR groups [34]. In comparison, the PARTNER study was the first to show substantial improvement in QoL at one-year follow-up after either TAVI or SAVR in high-risk elderly patients [37]. The baseline EQ-5D utility score increased by 14% to 0.66 at one-year post-TAVI. Fairbairn et al. demonstrated that QoL, as measured with the EQ-5D and EQ-VAS, improved early after TAVI and was maintained at one-year post-TAVI [38]. A review has shown a clear improvement in QoL after both TAVI and SAVR [39]. However, further research on QoL is needed.

### Limitations

FOOPAS has several limitations. The findings of our study are based on a single centre. Patient recruitment was difficult in many aspects, and the planned number of patients was not achieved. This was due to two main factors. First, it was particularly challenging to enrol patients in the conservative group, primarily due to a lack of motivation from the patient or their relatives to participate. Second, both recruitment and follow-up were affected by COVID-19 pandemic restrictions, resulting in a high dropout rate. The small conservative subgroup (*n* = 15) limited comparative analyses. Furthermore, patients with relevant cognitive impairment were excluded, reducing generalizability precisely to those frail individuals where geriatric input is most needed. Nevertheless, FOOPAS also has strengths, including in the prospective design.

The low number of outcome events restricts the power of multivariable models, increasing the risk of overfitting. Therefore, the reported associations should be interpreted as exploratory and hypothesis-generating rather than definitive.

## 5. Conclusions

FOOPAS showed that geriatric characteristics have no relevant impact on the treatment decision by heart teams in patients over 75 years of age with severe AS. This is despite the fact that these patients differed significantly in their functional and health status. Single geriatric assessment tools did not independently predict mortality; however, given the limited sample and exploratory design, absence of statistical significance does not imply lack of clinical relevance. Multidimensional scoring may hold greater promise for risk estimation.

Recent studies have confirmed that transcatheter aortic valve implantation (TAVI) achieves excellent outcomes in elderly patients, including those even older than 85 years of age, with survival rates comparable to those of younger cohorts and to surgical valve replacement. This leads to the conclusion that all cases of severe aortic valve stenosis should be treated, regardless of the advanced age of our patients. At the same time, comprehensive geriatric assessment has not yet demonstrated a consistent prognostic benefit in this setting, underscoring the need for randomized trials to clarify its role. Future multicenter studies should include cognitively impaired patients using proxy consent procedures to reflect real-world experience more accurately. These findings highlight both the durability and safety of TAVI in older populations and the ongoing uncertainty about integrating geriatric domains into structured decision-making.

## Figures and Tables

**Table 1:** Baseline characteristics.

Variable	Overall (*n* = 135, 100%)	SAVR (*n* = 39, 28.9%)	TAVI (*n* = 81, 60%)	Conservative (*n* = 15, 11.1%)	*p*-Value Groups ^1^	*p*-Value SAVR/TAVI ^2^
Gender (female)	61 (45%)	19 (48.7%)	34 (41.9%)	8 (53.3%)	0.626	0.486
Age (years)	81 (±4.6; 74–97)	78 (±2.4; 74–83)	82 (±4.3; 75–97)	87 (±4.7; 77–94)	<0.0001	<0.0001
**Echocardiography**						
AOVA cm^2^	0.7 (±0.2; 0–1.4)	0.8 (±0.2; 0.4–1.2)	0.7 (±2.1; 0–1.4)	0.7 (±0.2; 0.3–1)	0.494	0.475
AOV gradient max	69 (±27.6; 19–185)	72 (±24.4; 41–144)	68 (±29.1; 19–185)	70 (±26.4; 35–116)	0.491	0.235
AOV gradient mean	42 (±18.4; 12–112)	44 (±17.1; 20–97)	40 (±19.2; 12–112)	42 (±17; 19–81)	0.187	0.076
LVEF %	60 (±13.1; 20–85)	60 (±11.2; 26–85)	60 (±13.9; 20–84)	59 (±12.2; 34–81)	0.306	0.135
**Clinical presentation**						
NYHA class I	4 (2.9%)	1 (2.6%)	1 (1.2)	2 (13.3%)	0.0004	0.162
NYHA class II	43 (31.9%)	17 (43.6%)	26 (32.1%)	0 (0%)		
NYHA class III	74 (54.8%)	21 (53.8%)	46 (56.8%)	7 (46.7%)		
NYHA class IV	14 (10.4%)	0 (0%)	8 (9.9%)	6 (40%)		
Systolic BP (mmHg)	134 (±21.4; 96–193)	144 (±21.4; 104–177)	130 (±21; 96–183)	142 (±20.9; 110–180)	0.03	0.014
Diastolic BP (mmHg)	72 (±11.7; 46–104)	73 (±9.4; 57–104)	72 (±11.7; 46–101)	80 (±10.6; 60–90)	0.375	0.594
Heart rate/min	70 (±11.7; 48–115)	70 (±11.9; 56–115)	70 (±11; 48–99)	68 (±15.3; 52–99)	0.658	0.356
Respiratory rate/min	19 (±3.7; 12–30)	18.5 (±4; 12–26)	20 (±3.3; 12–28)	18 (±5.1; 12–30)	0.440	0.333
Oxygen saturation	96 (±2.3; 85–100)	97 (±2.35; 85–99)	96 (±2.3; 85–100)	95 (±1.9; 91–98)	0.075	0.273
BMI	26.8 (±4.9; 17.2–43.4)	26.9 (±4.6; 20.4–38.5)	27 (±5; 17.7–43.4)	23.4 (±5.1; 17.2–34.4)	0.1	0.942
**Laboratory tests**						
Hemoglobin g/dL	12.8 (±1.8; 8–17)	13.6 (±1.6; 9–17)	12.5 (±1.6; 8–16)	11.1 (±2.3; 8–15)	<0.0001	0.001
Sodium mmol/L	141 (±3.1; 127–149)	141 (±2.4; 134–147)	141 (±3; 127–147)	141 (±4.9; 132–149)	0.617	0.315
GFR mL/min	58 (±11.9; 5–60)	60 (±7; 34–60)	53 (±12.6; 5–50)	54 (±14.1; 19–60)	0.002	0.001
Pro-NT-BNP pg/mL	1636 (±10,242; 63–70,000)	758 (±4977; 63–30,028)	2145 (±12,028; 125–70,000)	2392 (±9850; 418–32,977)	0.007	0.004
CRP mg/dL	0.5 (±1.4; 0–12)	0.5 (±0.6; 0–4)	0.5 (±1; 0–7)	0.7 (±3.2; 0–12)	0.003	0.631
Albumin g/dL	4.1 (±0.4; 3–5)	4.2 (0.3; 4–5)	4.1 (±0.3; 3–5)	3.7 (±0.5; 3–5)	<0.0001	0.013
**Risk scores**						
EuroSCORE II	3.9 (±6.9; 1–61.4)	2.1 (±2.3; 1–12.6)	4.4 (±8; 1.4–61.4)	5.5 (±6.5; 1.6–29)	<0.0001	<0.0001
STS-Score	2.8 (±8.5; 0.6–88.5)	1.5 (±14.1; 0.6–88.5)	3.2 (±4.4; 1–18)	5.6 (±5.7; 1.8–17.9)	<0.0001	<0.0001

AOVA, aortic valve area; AOV, aortic valve; BMI, body mass index; BP, blood pressure; CRP, C-reactive protein; EuroSCORE II, European system for cardiac operative risk evaluation; GFR, glomerular filtration rate; LVEF, left ventricular ejection fraction; NYHA, New York Heart Associa-tion; STS-Score, Society of Thoracic Surgeons score. Gender, NYHA classes are shown as absolute numbers and percentages. Age, AOVA, AOV gradient max and mean, LVEF, systolic and diastolic BP, heart rate, respiratory rate, oxygen saturation, BMI, hemoglobin, sodium, GFR, pro-NT-BNP, CRP, Albumin, EuroSCORE II, and STS-Score are shown as median, standard deviation, and range. The *p*-value was calculated using Fisher’s exact test (for categorical data), Mann–Whitney U test ^2^, and Kruskali–Wallis test ^1^ (for comparing mean values).

**Table 2:** Geriatric assessment.

Variable	Overall (*n* = 135, 100%)	SAVR (*n* = 39, 28.9%)	TAVI (*n* = 81, 60%)	Conservative (*n* = 15, 11.1%)	*p*-Value Groups ^1^	*p*-Value SAVR/ TAVI ^2^
CIRS	11 (±3.8; 5–23)	10 (±3.3; 5–20)	12 (±3.6; 5–20)	15 (±4.9; 6–23)	0.002	0.006
Charlson-Index	3 (±1.8; 0–9)	2 (±1.7; 1–8)	3 (±1.6; 0–8)	3 (±2.3; 1–9)	0.005	0.001
Living independently	82 (60.7%)	32 (82.1%)	49 (60.5%)	1 (6.7%)	<0.0001	0.089
EQ-VAS	50 (±18.8; 10–100)	60 (±15.8; 25–90)	50 (±19.4; 10–100)	50 (±19.2; 15–95)	0.007	0.078
Frailty Score	1 (±1.5; 0–5)	1 (±0.9; 0–3)	2 (±1.5; 0–5)	3 (±1.5; 0–5)	<0.0001	0.0001
ADL	100 (±18.8; 10–100)	100 (±3.7; 85–100)	100 (±14.4; 30–100)	65 (±33; 10–100)	<0.0001	0.003
IADL	7 (±2.1; 0–8)	8 (±0.8; 5–8)	7 (±1.9; 1–8)	3 (±2.3; 0–7)	<0.0001	0.002
MMSE	29 (±3.2; 14–30)	29 (±1.4; 25–30)	28 (±2.9; 16–30)	25 (±4.8; 14–30)	<0.0001	0.004
GDS	2 (±2.4; 0–11)	2 (±1.8; 0–8)	2 (±2.5; 0–11)	3 (±2.7; 0–9)	0.68	0.077
Gaitspeed immobil	12 (8.9%)	1 (2.6%)	5 (6.2%)	6 (40%)	<0.0001	0.396
Gaitspeed sec	1 (±0.5; 0.1–2)	1.3 (±0.4; 0.5–2)	1 (±0.5; 0.2–2)	0.7 (±0.4; 0–1.4)	0.003	0.01
TUG immobil	16 (11.9%)	0 (0%)	9 (11.1%)	7 (46.7%)	<0.0001	0.03
TUG sec	11 (±10.6; 5–96)	10 (±14.1; 6–96)	12 (±7.5; 6–51)	15.5 (±13.8; 5–48)	0.001	0.026
PMS	9 (±2.8; 0–9)	9 (±1.5; 3–9)	8 (±2.6; 0–9)	2 (±3.4; 0–9)	<0.0001	0.001
Handgrip	26.8 (±10.4; 2.8–54.6)	29.8 (±9.6; 11.8–45.4)	28 (±10.4; 8.4–54.6)	20 (±9; 2.8–38.9)	0.015	0.227
MNA-SF	12 (±2.4; 5–14)	14 (±1.5; 9–14)	12 (±2.2; 5–14)	9 (±3; 5–14)	<0.0001	<0.0001
Medications	7 (±3; 0–16)	7 (±2.4; 2–12)	7 (±2.7; 0–14)	7 (±4.9; 0–16)	0.893	0.964

ADL, Activities of Daily Living; Charlson-Index, Charlson Comorbidity Index; CIRS, Cumulative Illness Rating Scale; EQ-VAS, EQ visual analogue scale; Frailty Score, Frailty Score based on Fried criteria; GDS, Geriatric Depression Scale; IADL, Instrumental Activities of Daily Living; MMSE, Mini-Mental-State-Examination; MNA-SF, Mini-Nutritional Assessment Short Form; PMS, Parker Mobility Score; TUG, Timed Up&Go. Living independently, Gaitspeed immobil, TUG immobil are shown as absolute numbers and percentages. CIRS, Charlson-Index, EQ-VAS, Frailty Score, ADL, IADL, MMSE, GDS, Gaitspeed, TUG, PMS, handgrip, MNA-SF, and medications are shown as median, standard deviation, and range. *p*-value was calculated using Fisher’s exact test (for categorical data), Mann–Whitney U test ^2^, and Kruskali–Wallis test ^1^ (for comparing mean values).

**Table 3:** Predictors of geriatric assessment after heart team’s decision between TAVI and SAVR (with the conservative group excluded because of small number).

Variable	OR (95% CI)	*p*-Value	R2 (Nagelkerke)
Age ^1^	1.43 (1.11–1.85)	0.006	0.586
EuroSCORE II ^1^	1.11 (0.85–1.47)	0.420	0.586
CIRS ^2^	1.01 (0.96–1.27)	0.189	0.427
EQ-VAS ^2^	1 (0.972–1.03)	0.978	0.412
ADL ^2^	0.94 (0.85–1.03)	0.154	0.434
IADL ^2^	0.74 (0.49–1.13)	0.166	0.430
Frailty Score ^2^	2.17 (1.02–4.63)	0.044	0.426
MMSE ^2^	0.7 (0.54–0.96)	0.026	0.471
GDS ^2^	1.1 (0.86–1.41)	0.439	0.417
Gaitspeed ^2^	0.62 (0.2–1.89)	0.396	0.400
PMS ^2^	0.93 (0.69–1.26)	0.632	0.414
Handgrip ^2^	1.02 (0.97–1.07)	0.442	0.417
MNA-SF ^2^	0.64 (0.48–0.86)	0.003	0.501

ADL, Activities of Daily Living; CIRS, Cumulative Illness Rating Scale; EQ-VAS, EQ visual analogue scale; Frailty Score, Frailty Score based on Fried criteria; GDS, Geriatric Depression Scale; IADL, Instrumental Activities of Daily Living; MMSE, Mini-Mental-State- Examination; MNA-SF, Mini-Nutritional Assessment Short Form; PMS, Parker Mobility Score; ^1^ binominal logistic regression adjusted for CIRS, EQ-VAS, ADL. IADL, Frailty-Score, MMSE, GDS, Gaitspeed, PMS, Handgrip and MNA-SF; ^2^ binominal logistic regression adjusted for age and EuroSCORE II.

**Table 4:** Multivariate analysis for TAVI vs. SAVR.

Variable	OR (95% CI)	*p*-Value	R2 (Nagelkerke)
Age ^1^	1.01 (0.76–1.32)	0.968	0.559
EuroSCORE II ^1^	1.58 (1.13–2.19)	0.007	0.559
CIRS ^2^	0.91 (0.72–1.15)	0.428	0.469
EQ-VAS ^2^	0.99 (0.95–1.05)	0.895	0.460
ADL ^2^	1.01 (0.97–1.05)	0.671	0.463
IADL ^2^	0.89 (0.62–1.28)	0.532	0.465
Frailty Score ^2^	1.38 (0.75–2.53)	0.299	0.476
MMSE ^2^	0.95 (0.76–1.2)	0.682	0.462
GDS ^2^	0.94 (0.68–1.29)	0.691	0.462
Gaitspeed ^2^	0.4 (0.06–2.84)	0.359	0.432
PMS ^2^	0.99 (0.74–1.36)	0.993	0.460
Handgrip ^2^	0.98 (0.91–1.08)	0.765	0.450
MNA-SF ^2^	1.02 (0.73–1.43)	0.891	0.460

ADL, Activities of Daily Living; CIRS, Cumulative Illness Rating Scale; EQ-VAS, EQ visual analogue scale; Frailty Score, Frailty Score based on Fried criteria; GDS, Geriatric Depression Scale; IADL, Instrumental Activities of Daily Living; MMSE, Mini-Mental-State-Examination; MNA-SF, Mini-Nutritional Assessment Short Form; PMS, Parker Mobility Score; ^1^ binominal logistic regression adjusted for CIRS, EQ-VAS, ADL, IADL, Frailty Score, MMSE, GDS, Gaitspeed, PMS, Handgrip and MNA-SF; ^2^ binominal logistic regression adjusted for age and EuroSCORE II.

**Table 5:** Baseline geriatric assessments and post-intervention QoL.

Variable	Overall (*n* = 88, 100%)	Increase in QoL (*n* = 44, 50%)	No Increase in QoL (*n* = 44, 50%)	*p*-Value
CIRS	10 (±3.7; 5–20)	11.5 (±3.8; 6–20)	10 (±3.6; 5–20)	0.182
Charlson-Index	3 (±1.7; 1–8)	3 (±1.5; 1–7)	3 (±1.8; 1–8)	0.805
Living independently	62 (70.5%)	33 (75%)	29 (65.9%)	0.332
EQ-VAS	60 (17.7; 10–100)	50 (±15.6; 10–90)	72 (±16; 50–100)	<0.0001
Frailty Score	1 (±1.4; 0–5)	1 (±1.3; 0–4)	1 (±1.5; 0–5)	0.607
ADL	100 (±10.6; 30–100)	100 (±13.3; 30–100)	100 (±6.7; 65–100)	0.777
IADL	8 (±1.6; 1–8)	8 (±1.7; 1–8)	7 (±1.4; 2–8)	0.082
MMSE	29 (±2.4; 19–30)	29 (±2.3; 19–30)	29 (±2.6; 19–30)	0.574
GDS	2 (±2.5; 0–11)	2 (±2.8; 0–11)	2 (2.2. (0–8)	0.959
Gaitspeed immobil	0 (0%)	0 (0%)	0 (0%)	-
Gaitspeed m/s	1.1 (±0.5; 0.2–2)	1.1 (±0.5; 0.3–2)	1.1 (±0.5; 0.2–2)	0.993
TUG immobil	4 (4.5%)	3 (6.8%)	1 (2.3%)	0.616
TUG sec	11 (±6.8; 6–51)	11 (±6.4; 6–44)	11 (±7.3; 7–51)	0.292
PMS	9 (±2.1; 2–9)	9 (±2.2; 2–9)	9 (±1.9; 2–9)	0.618
Handgrip	28.6 (±10.1; 11.6–54.6)	29.8 (±9.8; 11.6–54.6)	27.3 (±10.5; 13.2–46.3)	0.936
MNA-SF	12 (±2.1; 6–14)	12 (±2.3; 6–14)	12 (±1.8; 7–14)	0.819
Medications	7 (±2.6; 0–12)	7 (±2.2; 2–11)	7 (±2.9; 0–12)	0.807

ADL, Activities of Daily Living; Charlson-Index, Charlson Comorbidity Index; CIRS, Cumulative Illness Rating Scale; EQ-VAS, EQ visual analogue scale; Frailty Score, Frailty Score based on Fried criteria; GDS, Geriatric Depression Scale; IADL, Instrumental Activities of Daily Living; MMSE, Mini-Mental-State-Examination; MNA-SF, Mini-Nutritional Assessment Short Form; PMS, Parker Mobility Score; TUG, Timed Up&Go. Living independently, Gaitspeed immobil, TUG immobil are shown as absolute numbers and percentages. CIRS, Charlson-Index, EQ-VAS, Frailty Score, ADL, IADL, MMSE, GDS, Gaitspeed, TUG, PMS, handgrip, MNA-SF, and medications are shown as median, standard deviation, and range. *p*-value was calculated by using Fisher’s exact test (for categorical data) and Mann–Whitney U test (for comparing median values).

## Data Availability

The data presented in this study are available on request from the first authors. The data are not publicly available due to date protection regulations.

## Abbreviations

The following abbreviations are used in this manuscript:

ADL	Activities of Daily Living
AOVA	Aortic Valve Area
AOV	Aortic Valve
BMI	Body Mass Index
BP	Blood Pressure
Charlson-Index	Charlson Comorbidity Index
CIRS	Cumulative Illness Rating Scale
EQ-VAS	EQ Visual Analogue Scale
EuroSCORE II	European System for Cardiac Operative Risk Evaluation
Frailty Score	Frailty Score based on Fried Criteria
GDS	Geriatric Depression Scale
GFR	Glomerular Filtration Rate
IADL	Instrumental Activities of Daily Living
LVEF	Left Ventricular Ejection Fraction
NYHA	New York Heart Association
MMSE	Mini-Mental-State-Examination
MNA-SF	Mini-Nutritional Assessment Short Form
PMS	Parker Mobility Score
TAVI	Transcatheter Aortic Valve Implantation
TUG	Timed Up&Go
SAVR	Surgical Aortic Valve Implantation
STS-Score	Society of Thoracic Surgeons score

## Appendix A

**Table A1:** Baseline characteristics and one-year-mortality.

Variable	Survivors (*n* = 100, 100%)	Non-Survivors (*n* = 11, 27.9%)	*p*-Value
Gender (female)	43 (43%)	3 (27.3%)	0.357
Age (years)	81 (±4.2; 75–97)	84 (±4.9; 77–92)	0.050
AOVA cm^2^	0.7 (±0.2; 0–1.4)	0.7 (±0.14; 0.5–1)	0.652
AOV gradient max	71 (±27.8; 19–185)	62 (±18.1; 27–92)	0.020
AOV gradient mean	42 (±18.7; 12–112)	40 (±11.9; 15–52)	0.126
LVEF %	60 (±12.2; 26–84)	55 (±18.4; 20–68)	0.119
NYHA class I	3 (3%)	0 (0%)	0.211
NYHA class II	38 (38%)	1 (9.1%)	
NYHA class III	50 (50%)	8 (72.7%)	
NYHA class IV	9 (9%)	2 (18.2%)	
Systolic BP mmHG	135 (±19.6; 99–180)	115 (±18.6; 100–165)	0.003
Diastolic BP mmHG	72 (±11; 46–104)	65 (±11.9; 49–90)	0.022
Heart rate/min	70 (±12.2; 48–115)	68 (±13.7; 58–99)	0.767
Respiratory rate/min	20 (±3.07; 12–30)	18 (±3.5; 14–26)	0.839
Oxygen saturation	97 (±2.2; 85–100)	96 (±3.7; 85–99)	0.385
BMI	27.2 (±4.8; 17.2–43)	23.4 (±19.4–26.7)	0.001
Hemoglobin g/dL	12.9 (±1.6; 8–17)	11.4 (±2; 8–14)	0.005
Sodium mmol/L	141 (±3.1; 127–149)	142 (±2.1; 137–144)	0.605
GFR mL/min	58 (±11.6; 5–60)	47 (±14.1; 19–60)	0.030
Pro-NT-BNP pg/mL	1335 (±10,835; 63–70,000)	4395 (±11,153; 645–37,624)	0.012
CRP mg/dL	0.5 (±1.1; 1–7)	0.5 (±0.3; 1–1)	0.264
Albumin g/dL	4.1 (±0.3; 3–5)	3.8 (±0.4; 3–4)	0.011
EuroSCORE II	3.2 (±4; 1–22.8)	14.4 (±16.2; 3.1–61.4)	<0.0001
STS-Score	2.5 (±9.4; 0.6–88.5)	5.6 (±6; 1.9–19)	<0.002
CIRS	10.5 (±3.9; 5–21)	13 (±4.3; 7–23)	0.258
Charlson-Index	3 (±1.8; 0–8)	5 (±1.9; 2–9)	0.004
Living independently	69 (69%)	3 (27.3%)	0.001
EQ-VAS	55 (17.4; 15–92)	50 (±20.9; 10–80)	0.045
Frailty Score	1 (±1.4; 0–5)	3 (1.5; 0–5)	0.002
ADL	100 (±15; 10–100)	85 (±27.5; 15–100)	0.011
IADL	8 (±1.8; 0–8)	5 (±2.1; 2–8)	0.002
MMSE	29 (±3; 14–30)	28 (±2.1; 23–29)	0.029
GDS	2 (±2.4; 0–9)	3 (±1.8; 0–7)	0.141
Gaitspeed immobil	2 (2%)	3 (27.3%)	0.007
Gaitspeed m/s	1 (±0.5; 0.2–2)	0.7 (±0.24; 0.6–1.3)	0.035
TUG immobil	5 (5%)	3 (27.3%)	0.031
TUG sec	11 (±7.1; 5–51)	18.5 (±6.1; 7–27)	0.012
PMS	9 (±2.4; 0–9)	4 (±2.9; 0–9)	0.001
Handgrip	27.6 (±10.3; 2.8–54.6)	22.8 (±8.7; 8.4–40)	0.066
MNA-SF	12 (±2.3; 5–14)	11 (±2.3; 6–14)	0.039
Medications	7 (±2.7; 0–12)	7 (±2.9; 5–14)	0.119

ADL, Activities of Daily Living; AOVA, aortic valve area; AOV, Aortic valve; BMI, body mass index; BP, blood pressure; Charlson-Index, Charlson Comorbidity Index; CIRS, Cumulative Illness Rating Scale; EQ-VAS, EQ visual analogue scale; EuroSCORE II, European system for cardiac operative risk evaluation; Frailty Score, Frailty Score based on Fried criteria; GDS, Geriatric Depression Scale; GFR, glomerular filtration rate; IADL, Instrumental Activities of Daily Living; LVEF, left ventricular ejection fraction; NYHA, New York Heart Association; MMSE, Mini-Mental-State-Examination; MNA-SF, Mini-Nutritional Assessment Short Form; PMS, Parker Mobility Score; TUG, Timed Up&Go; STS-Score, Society of Thoracic Surgeons score. Gender, NYHA, Living independently, Gaitspeed immobil, TUG immobil are shown as absolute numbers and percentage. All others are shown as median, standard deviation and range. *p*-value was calculated by using Fisher’s exact test and the Pearson-Chi-Quadrat (for categorical data) and the Mann–Whitney U test (for comparing mean values).

**Table A2:** Baseline characteristics and post-intervention QoL.

Variable	Overall (*n* = 88, 100%)	Increase of QoL (*n* = 44, 50%)	No Increase of QoL (*n* = 44, 50%)	*p*-Value
Gender (female)	36 (40.9%)	17 (38.6%)	19 (43.2)	0.829
Age (years)	80 (±3.5; 75–89)	80 (±3.7; 75–89)	81 (±3.3; 75–89)	0.206
TAVI (yes)	61 (69.3%)	31 (70.5%)	30 (68.2%)	1.000
Echocardiography				
AOVA cm^2^	0.7 (±0.2; 0–1.4)	0.7 (±2.2; 0–1.2)	0.7 (±2.2; 0–1.4)	0.514
AOV gradient max	70 (±27.3; 25–185)	70 (±21.5; 26–128)	70 (±32.2; 25–185)	0.735
AOV gradient mean	42 (±18.3; 16–112)	42 (±14.5; 16–83)	42 (±21.7; 16–112)	0.835
LVEF %	60 (±12.4; 25–84)	60 (±14; 25–79)	60 (±10.6; 26–84)	0.906
Clinical presentation				
NYHA class I	2 (2.3%)	0 (0%)	2 (4.5%)	0.101
NYHA class II	37 (42%)	19 (43.2%)	18 (40.9%)	
NYHA class III	45 (51.1%)	21 (47.7%)	24 (54.5%)	
NYHA class IV	4 (4.5%)	4 (9.1%)	0 (0%)	
Systolic BP mmHG	133 (±21.1; 99–183)	132 (±20.7; 100–183)	133.5 (±21.7; 99–180)	0.605
Diastolic BP mmHG	72.5 (±11; 46–104)	75.5 (±12.1; 50–1049	70.5 (±9.9; 46–91)	0.410
Heart rate/min	69.5 (±11; 48–115)	70.5 (±9.1; 52–89)	68 (±12.7; 48–115)	0.390
Respiratory rate/min	20 (±3.6; 12–28)	18 (±3.7; 12–28)	20 (±3.4; 12–26)	0.205
Oxygen saturation	97 (±2.2; 85–100)	96 (±1.7; 91–99)	97 (±2.5; 85–100)	0.117
BMI	27.9 (±4.7; 20.2–43.4)	28.3 (±4.9; 20.3–43.4)	26.8 (±4.5; 20.2–42.2)	0.127
Laboratory tests				
Hemoglobin g/dL	12.9 (±1.6; 9–17)	13 (±1.6; 9–16)	12.9 (±1.7; 10–17)	0.710
Sodium mmol/L	141 (±2.9; 127–147)	141 (±3.4; 127–147)	141 (±2.4; 137–147)	0.242
GFR mL/min	58.5 (±10.9; 5–60)	56.5 (±11.5; 5–60)	60 (±10.4; 12–60)	0.497
Pro-NT-BNP pg/mL	1353 (±91,710; 63–70,000)	1713 (±11,196; 77–70,000)	1170 (±6410; 63–30,028)	0.433
CRP mg/dL	0.5 (±0.8; 1–7)	0.5 (±1.3; 1–7)	0.5 (±0.3; 1–2)	0.465
Albumin g/dL	4.2 (±0.3; 3–5)	4.1 (±0.3; 3–5)	4.2 (±0.3; 4–5)	0.376
Risk scores				
EuroSCORE II	3.2 (±4.2; 1–22.94)	3.9 (±4.5; 1–17.4)	2.9 (±3.8; 1.1–22.8)	0.137
STS-Score	2.4 (±9.6; 0.6–88.5)	2.5 (±2.8; 0.6–15.2)	2.4 (±13.5; 0.9–88.5)	0.986

AOVA, aortic valve area; AOV, Aortic valve; BMI, body mass index; BP, blood pressure; CRP, C-reactive protein; EuroSCORE II, European system for cardiac operative risk evaluation; GFR, glomerular filtration rate; LVEF, left ventricular ejection fraction; NYHA, New York Heart Association; STS-Score, Society of Thoracic Surgeons score. Gender, NYHA classes are shown as absolute numbers and percentage. Age, AOVA, AOV gradient max and mean, LVEF, systolic and diastolic BP, heart rate, respiratory rate, oxygen saturation, BMI, hemoglobin, sodium, GFR, pro-NT-BNP, CRP, Albumin, EuroSCORE II and STS-Score are shown as median, standard deviation and range. *p*-value was calculated by using Fisher’s exact test (for categorical data) and Mann–Whitney U test (for comparing median value).

**Table A3:** Quality of life results for each component.

	A1				A3				A4			
Item	Overall *n* = 135	AVR *n* = 39	TAVI *n* = 81	Conservative *n* = 15	Overall *n* = 100	AVR *n* = 30	TAVI *n* = 64	Conservative *n* = 6	Overall *n* = 92	AVR *n* = 27	TAVI *n* = 60	Conservative *n* = 4
M1 M2 M3	65 (48.1%) 64 (47.4%) 6 (4.4%)	25 (64.1%) 14 (35.9%) 0 (0%)	34 (42%) 45 (55.6%) 2 (2.5%)	6 (40%) 5 (33.3%) 4 (26.7%)	57 (57%) 43 (43%) 0 (0%)	23 (76.7%) 7 (23.3%) 0 (0%)	32 (50%) 32 (50%) 0 (0%)	0 (0%) 2 (33.3%) 4 (66.7%)	43 (46.7%) 48 (52.2%) 1 (1.1%)	17 (63%) 10 (37%) 0 (0%)	25 (41.7%) 35 (58.3%) 0 (0%)	1 (25%) 2 (50%) 1 (25%)
SC1 SC2 SC3	106 (78.5%) 23 (17%) 6 (4.4%)	39 (100%) 0 (0%) 0 (0%)	60 (74.1%) 18 (22.2%) 3 (3.7%)	7 (46.7%) 5 (33.3%) 3 (20%)	85 (85%) 14 (14%) 1 (1%)	29 (96.7%) 1 (3.3%) 0 (0%)	52 (81.3%) 18 (18.8%) 0 (0%)	4 (66.7%) 1 (16.7%) 1 (16.7%)	71 (77.2%) 18 (19.6%) 3 (3.3%)	25 (92.6%) 2 (7.2%) 0 (0%)	44 (73.3%) 14 (23.3%) 2 (3.3%)	2 (50%) 1 (25%) 1 (25%)
UA1 UA2 UA3	78 (57.8%) 43 (31.9%) 14 (10.4%)	32 (82.1%) 6 (15.4%) 1 (2.6%)	40 (49.4%) 34 (42%) 7 (8.6%)	6 (40%) 3 (20%) 6 (40%)	72 (72%) 28 (28%) 0 (0%)	25 (83.3%) 5 (16.7%) 0 (0%)	43 (67.2%) 21 (32.8%) 0 (0%)	4 (66.7%) 2 (33.3%) 0 (0%)	64 (69.4%) 24 (26.1%) 4 (4.3%)	22 (81.5%) 5 (18.5%) 0 (0%)	40 (66.7%) 17 (28.3%) 3 (5%)	1 (25%) 2 (50%) 1 (25%)
PD1 PD2 PD3	43 (31.9%) 65 (48.1%) 27 (20%)	15 (38.5%) 20 (51.3%) 4 (10.3%)	24 (29.6%) 39 (48.1%) 18 (22.2%)	4 (26.7%) 6 (40%) 5 (33.3%)	32 (32%) 55 (55%) 13 (13%)	14 (46.7%) 15 (50%) 1 (3.3%)	16 (25%) 37 (57.8%) 11 (17.2%)	2 (33.3%) 3 (50%) 1 (16.7%)	34 (37%) 49 (53.3%) 9 (9.8%)	11 (40.7%) 14 (51.9%) 0 (0%)	21 (35%) 32 (53.3%) 7 (11.7%)	2 (50%) 2 (50%) 0 (0%)
AD1 AD2 AD3	95 (70.4%) 35 (25.9%) 5 (3.7%)	26 (66.7%) 12 (30.8%) 1 (2.6%)	59 (72.8%) 20 (24.7%) 2 (2.5%)	10 (66.7%) 3 (20%) 2 (13.3%)	82 (82%) 16 (16%) 2 (2%)	25 (83.3%) 5 (16.7%) 0 (0%)	52 (81.3%) 10 (15.6%) 2 (3.1%)	5 (83.3%) 1 (16.7%) 0 (0%)	75 (81.5%) 14 (15.2%) 3 (3.3%)	21 (77.8%) 6 (22.2%) 0 (0%)	49 (81.7%) 8 (13.3%) 3 (5%)	4 (100%) 2 (50%) 2 (50%)
YHT	50 (±18.8)	60 (±18.8)	50 (±19.4)	50 (±19.2)	72 (±16.3)	80 (±17.4)	70 (±15.4)	53 (±9.6)	70 (±17.7)	75 (±17.8)	70 (±17.7)	60 (±10.3)

Mobility: M1, I have no problems in walking about; M2, I have some problems in walking about; M3, I am confined to bed; Self-care: SC1, I have no problems with self-care; SC2, I have some problems washing or dressing myself; SC3, I am unable to wash or dress myself; Usual Activities: UA1, I have no problems with performing my usual activities; UA2, I have some problems with performing my usual activities; UA3, I am unable to perform my usual activities; Pain/Discomfort: PD1, I have no pain or discomfort; PD2, I have moderate pain or discomfort; PD3, I have extreme pain or discomfort; Anxiety/Depression: AD1, I am not anxious or depressed; AD2, I am moderately anxious or depressed; AD3, I am extremely anxious or depressed; YHT, Your health today.
